# Transcriptome analysis in non-model species: a new method for the analysis of heterologous hybridization on microarrays

**DOI:** 10.1186/1471-2164-11-344

**Published:** 2010-05-31

**Authors:** Cyril Degletagne, Céline Keime, Benjamin Rey, Marc de Dinechin, Fabien Forcheron, Paul Chuchana, Pierre Jouventin, Christian Gautier, Claude Duchamp

**Affiliations:** 1 Université de Lyon, F-69000, Lyon; Laboratoire de Physiologie Intégrative, Cellulaire et Moléculaire, UMR 5123 CNRS - Université Lyon 1, 43 Bvd 11 Novembre 1918, F-69622 Villeurbanne Cedex, France; 2 Pôle Rhône Alpes de Bioinformatique, Université Lyon 1, Bâtiment Gregor Mendel, 16 rue Raphaël Dubois, 69622 Villeurbanne cedex, France; 3 Université de Lyon, F-69000, Lyon; Laboratoire de Biométrie et Biologie Evolutive, UMR 5558 CNRS - Université Lyon 1, 43 Bvd 11 Novembre 1918, F-69622 Villeurbanne Cedex, France; 4 UMR 5175 Centre d'Ecologie Fonctionnelle et Evolutive - CNRS, 1919 route de Mende 34293 Montpellier CEDEX 5, France; 5 UMR 2724 Génétique et Évolution des Maladies Infectieuses - CNRS-IRD, 911 avenue Agropolis, 34394 Montpellier Cedex 5, France; 6 CIRAD UMR 17 [UMR 177 IRD-CIRAD], TA A-17/G, Campus International de Baillarguet, 34398 Montpellier CEDEX 5, France; 7 U844, 80 avenue Augustin Fliche F-34295 Montpellier, France

## Abstract

**Background:**

Recent developments in high-throughput methods of analyzing transcriptomic profiles are promising for many areas of biology, including ecophysiology. However, although commercial microarrays are available for most common laboratory models, transcriptome analysis in non-traditional model species still remains a challenge. Indeed, the signal resulting from heterologous hybridization is low and difficult to interpret because of the weak complementarity between probe and target sequences, especially when no microarray dedicated to a genetically close species is available.

**Results:**

We show here that transcriptome analysis in a species genetically distant from laboratory models is made possible by using MAXRS, a new method of analyzing heterologous hybridization on microarrays. This method takes advantage of the design of several commercial microarrays, with different probes targeting the same transcript. To illustrate and test this method, we analyzed the transcriptome of king penguin pectoralis muscle hybridized to Affymetrix chicken microarrays, two organisms separated by an evolutionary distance of approximately 100 million years. The differential gene expression observed between different physiological situations computed by MAXRS was confirmed by real-time PCR on 10 genes out of 11 tested.

**Conclusions:**

MAXRS appears to be an appropriate method for gene expression analysis under heterologous hybridization conditions.

## Background

During the last decade, the use of DNA microarrays has become a key tool in molecular biology. This technology is commonly used for physiological and medical studies to generate snapshots of gene expression patterns in tissues of organisms exposed to different environmental conditions, allowing us to infer regulatory pathways involved in cellular responses to these conditions. The increased prevalence of microarray technology has benefited from the emergence of easily available commercial arrays. However, commercial microarrays target a limited number of species. Moreover, for many non-traditional model organisms, the insufficient amount of sequence data prevents the development of dedicated microarrays. Therefore, a few studies have investigated the use of heterologous array hybridization, [i.e. hybridization on arrays designed for a particular species (hereafter called the reference species) to explore modifications of gene expression patterns of another species (hereafter called the studied species)] and highlighted the difficulties inherent to this approach.

Heterologous hybridization is usually considered a non-standard utilization of microarrays [1]. Indeed, it raises a number of difficulties, essentially due to the sequence divergence between the reference and the studied species [2]. A major consequence of heterologous hybridization is a global reduction of hybridization fluorescence signal ([1] and references therein). This reduction artificially decreases the number of differentially expressed genes detected by standard statistical tests, leading to a misrepresentation of the variation in transcriptomic profiles ([1] and references therein). Another issue of heterologous hybridization is cross-hybridization [3]. Indeed, microarrays are designed so that each probe is specific to one transcript sequence in the dedicated species. However, this specificity is not guaranteed when transcripts from another species are hybridized onto the array. On the other hand, the use of heterologous hybridization does not amplify the problem of differentiating paralog expression levels compared to the use of the dedicated platform species.

For all these reasons, the use of heterologous hybridization should be preceded by a careful choice of the type of microarray to use and followed by an appropriate analysis of the results.

To choose the most appropriate microarray to use, one has to select the model organism with the lowest sequence divergence from the studied species [4]. Due to the lack of sufficient sequence data for all studied species, expression profiling results are the most robust when using microarrays dedicated to the reference species with the smallest phylogenetic distance from the studied species [5].

Once the reference species is chosen, one has to choose the best type of probe to use: either short oligonucleotide probes, such as those on Affymetrix GeneChips^®^, or longer probes, such as long oligomers or even full-length cDNAs. Microarrays with long probes might be less sensitive to sequence mismatches and thus facilitate heterologous hybridization [1-3,6]. However, most arrays with long probes contain only one probe per transcript. It can be advantageous to use arrays with several short probes targeting the same transcript: the sequence of some probes may be more similar to the orthologous sequence in the species of interest than others. Therefore, one can consider only those the probes that recognize conserved areas of genes between reference and studied species [3,7,8]. These specific probes can be determined from sequence comparison [3,8] or experimentally after hybridizing genomic DNA to the microarray [7]. However, the lack of sufficient sequence data in many species prevents the determination by sequence comparison, and the hybridization of genomic DNA raises the problem of setting the threshold of fluorescence to accept or reject the information provided by a probe [7].

In the present study, we were interested in gene expression changes in the pectoralis muscle of juvenile king penguins at a key step of their development, during the transition from terrestrial to marine life. Strictly terrestrial during their first year after hatching, king penguin chicks must then depart to sea to become self-sufficient, and pectoralis muscle is largely involved in penguin adaptation to the marine environment [9]. We choose the chicken as our reference species, as this is the closest model species for which microarrays are available. Chicken and king penguin are separated by approximately 100 millions years of phylogenetic divergence [10]. We decided to use Affymetrix GeneChip^® ^Chicken Genome Arrays because they present on average 11 different probe pairs per probe set (i.e., a set of perfect-match and mismatch probes targeting one given transcript), which should increase the probability that at least one probe will hybridize with the heterologous transcript. We then developed a new method (MAXRS, for maximum rank sum) to analyze heterologous hybridization transcriptomic profiles. This method takes advantage of the design of Affymetrix microarrays with different probes targeting the same transcript. Statistical analyses were then conducted to identify differentially expressed genes in the pectoralis muscle between never-immersed and sea-acclimated penguins. Finally, we confirmed by quantitative PCR the expression profiles of 10 up- or down-regulated genes exhibiting a wide range of fold changes, out of 11 tested. MAXRS therefore appears to be an appropriate method of gene expression analysis under heterologous hybridization conditions and provides new perspectives in the application of microarray technology to ecological physiology studies.

## Results and Discussion

### Heterologous hybridization

Two sets of juvenile king penguins (*Aptenodytes patagonicus*) were captured at different degrees of acclimation to marine life. In the first group, four penguins were captured just before they underwent their first immersion in cold sea water (thereafter called NI for never-immersed), while the second group was composed of three penguins that had completely accomplished their acclimation to marine life (thereafter called SA for sea-acclimated). For each of these penguins, an Affymetrix GeneChip^® ^Chicken Genome Array was hybridized with RNA from a pectoralis muscle biopsy.

### Global characterization of the fluorescence signal

**Figure 1:**
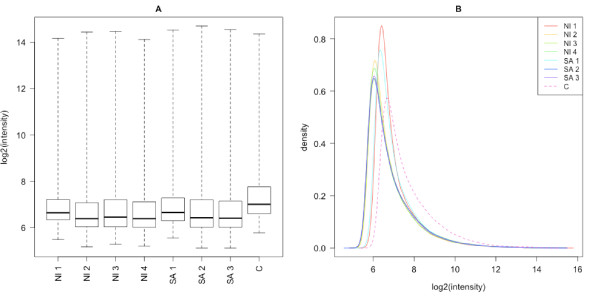
**Comparison of the distribution of fluorescence in microarrays with homologous (C, chicken) or heterologous (NI1 to NI4, never-immersed penguins and SA1 to SA3, sea-acclimatized penguins) hybridizations**. All samples were hybridized on Affymetrix GeneChip^® ^Chicken Genome Arrays. Data for the homologous hybridization (C) were downloaded from the Gene Expression Omnibus (GSM157808). A: Fluorescence intensity boxplot. B: Fluorescence intensity density plot.

Figure 1 compares the distribution of signal intensities in our arrays with those on the same type of array hybridized with chicken cRNA. This latest dataset corresponds to public gene expression data downloaded from the Gene Expression Omnibus (GSM157808). The same figure also appears with other public chicken microarray datasets: the fluorescence signal on our arrays hybridized with penguin RNA is relatively low compared with arrays hybridized with chicken RNA, as expected. Thus, the mean fluorescence intensity is lower in heterologous than in homologous hybridization, as previously documented in [1,3,5] for other species.

This probably results from the sequence divergence between chicken and penguins, that diverged approximately 100 Myr ago [10]. Very few penguin sequences have already been published, but the comparison of these sequences with the orthologous chicken sequences gave us a first estimation of the sequence divergence between these two species: we found between 89.4% and 91.7% identity between these sequences (see Additional file 1).

After these general considerations, we will describe the method we designed to analyze our heterologous hybridization data (we will hereafter only consider these data in our analysis).

### Characterization of the probes with fluorescence intensity above background

To determine which probe signal was sufficient to be exploited as a measure of gene expression, we considered the intensities of the spots located in the region of the array without any probe as a measure of the background intensity distribution. Only 40% of the spots corresponding to perfect match probes had fluorescence intensity above the 95^th ^percentile of the background intensity distribution (hereafter called the background level). As the global fluorescence signal intensity was low, we did not take into account the mismatch probes in our analysis. Therefore, we further call a probe set the collection of perfect-match probes targeting one given transcript, and the perfect-match probes will hereafter be referred to as probes. Additionally, we considered only the probes with a fluorescence intensity above the background level in at least one of the microarrays analyzed (this corresponded to 171,384 probes belonging to 36,897 probe sets).

**Figure 2:**
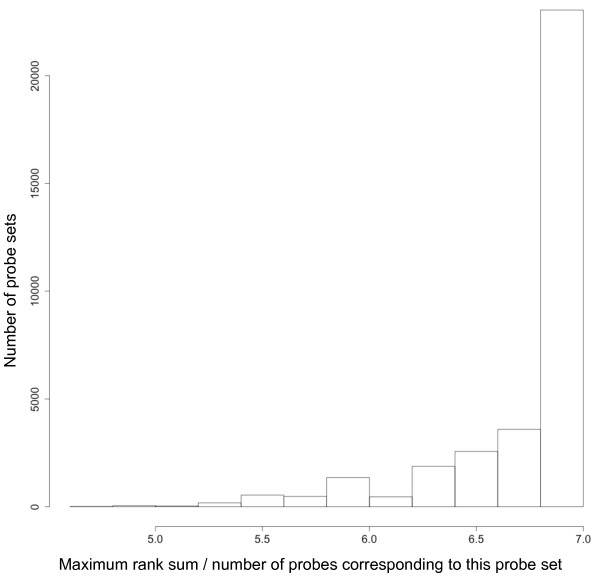
**For a given probe set, the same probe had the largest fluorescence intensity in the majority of arrays**. This figure represents the distribution of the maximum rank sum of the probes in each probe set divided by the number of probes corresponding to this probe set. If, for a given probe set, the same probe had the highest fluorescence intensity in all 7 microarrays considered here, we expected that this maximum rank sum divided by the number of probes would equal 7.

We took advantage of the design of Affymetrix arrays, with, on average, 11 probes per probe set and compared the fluorescence intensity of all probes belonging to the same probe set. For most of the probe sets, at least one probe had a relatively high signal: we found that 96% of the probe sets had at least one corresponding probe with a signal above the background level (corresponding to 36,897 out of the 38,536 probe sets of the array). Moreover, if we ranked probes belonging to a given probe set according to their fluorescence intensity, these rankings were similar among slides for the vast majority (94%) of the probe sets (Friedman p-value < 0.05). For a given probe set, the same probe had the greatest fluorescence intensity in the majority of arrays (Figure 2). We denote p = 1..P_t _the different probes belonging to a given probe set, m = 1..M the microarrays analyzed and  the rank sum of a probe p from a probe set t in all microarrays analyzed. This figure represents the distribution of  for all probe sets. If, for a given probe set, the same probe has the highest fluorescence intensity in all seven microarrays considered, we expect that  = 7. This figure therefore indicates that heterologous hybridizations are highly reproducible, even if the microarray is dedicated to a phylogenetically distant species. These observations led us to develop the MAXRS method.

### The maximum rank sum (MAXRS) method

This method is based on the observation that for the majority of the probe sets, the same probe had the highest fluorescence intensity in almost all arrays. We therefore hypothesized that among the different probes belonging to a probe set, the one with the greatest intensity should target the most conserved region between chicken and penguin mRNA. This probe should be the most appropriate to study the expression level of the penguin gene. Therefore, the MAXRS method consists in determining the probe with the highest fluorescence intensity in most microarrays. More precisely:

1. For each microarray m = 1..M and for each probe set t = 1..T, we sort the fluorescence intensity values on microarray m of all probes p = 1..P_t _belonging to the probe set t in increasing order. We denote by r_mtp _these ranks.

2. For each probe set t = 1..T and for each probe p = 1..P_t _belonging to the probe set t, we calculate the rank sum of this probe in all microarrays: .

3. For each probe set t = 1..T, we keep the probe p with the highest RS_tp_. If several probes have the same RS_tp _and this is the highest one, we keep the probe with the highest mean fluorescence intensity on all microarrays. The intensity of the selected probe on all microarrays is therefore used as an estimator of the expression of the gene represented by the probe set t.

**Figure 3:**
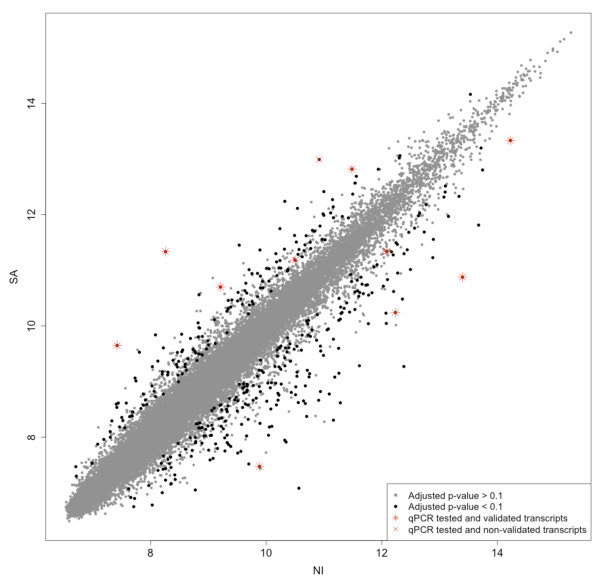
**Scatter plot comparing gene expression between penguins before (NI) and after sea acclimation (SA)**. Each point represents the mean expression level of a gene in NI and SA conditions. Black dots represent differentially expressed genes between both situations. Red symbols represent the differentially expressed genes tested by qPCR: red stars correspond to the validated genes and red crosses to non-validated ones.

We then normalized the data to make them comparable across microarrays and searched for differentially expressed genes by using the empirical Bayes moderated t-statistics proposed by Smyth [11]. We then used the method proposed by Benjamini and Hochberg [12] to ensure a false discovery rate of 10%. This led us to identify 240 significantly up-regulated and 154 down-regulated genes in pectoralis muscle of penguin juveniles after their acclimation to marine life (Figure 3).

We finally compared the results of the MAXRS method with results obtained by the Affymetrix software GCOS (GeneChip Operating Software). For this purpose, we applied the statistical test described above to the data obtained by GCOS. This resulted in the identification of 40 significantly up-regulated and 21 down-regulated genes.

### Validation of the differentially expressed genes

**Figure 4:**
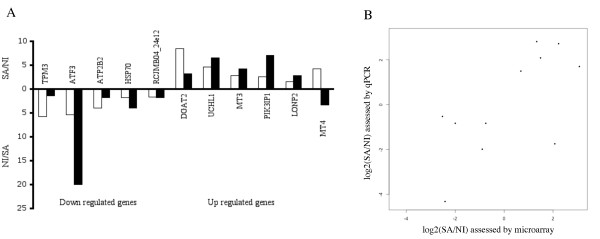
**Comparison of the gene expression differences assessed by our heterologous hybridization analysis and by qPCR**. A: Expression fold changes of the 11 genes tested by quantitative PCR. These fold changes correspond to SA/NI for the genes up-regulated during the transition from terrestrial to marine life (represented above the x-axis), and to NI/SA for the down-regulated genes (represented below the x-axis). The white bars correspond to the fold changes assessed by microarray and the black bars to the fold changes assessed by quantitative PCR. B: Comparison of the fold changes assed by microarray and by qPCR. Pearson correlation coefficient = 0.68 (p-value = 0.02).

We first quantified by quantitative PCR (qPCR) the relative expression levels of significantly differentially expressed genes from the MAXRS method, exhibiting various gene expression levels and fold changes (see Figure 3; the fold change of the selected genes varied from 1.6 to 8.4). For 10 out of 11 tested genes, qPCR confirmed the microarray results concerning the direction of gene expression variation, even for weak gene expression changes (Figure 4). As we do not know the penguin sequence of these mRNA, the qPCR primers were designed against chicken mRNA sequences. However, we confirmed those variations using penguin-specific primers we designed for six mRNA sequences that we sequenced (see Additional file 2). For the validated genes, the fold change assessed by qPCR was not always the same as assessed by the microarrays, and the change was more often higher with qPCR than with microarrays, as previously described for homologous hybridization [13,14].

We then quantified by qPCR the relative expression levels of several significantly differentially expressed genes from the GCOS Affymetrix method. The direction of gene expression variation was confirmed for four out of six tested genes (see Additional file 3). Considering this rate of validation and the small number of differentially expressed genes from GCOS, our method seems to be more sensitive and more specific than GCOS. Actually, the GCOS algorithm used the 11 probe pairs (perfect-match and mismatch probes) of each probe set to evaluate the expression of each gene. Considering cross-hybridization and the effect of sequence divergence, the use of GCOS is clearly not suitable for heterologous hybridization analyses.

The differentially expressed genes from MAXRS allowed us to highlight the onset of biologically meaningful physiological pathways. Indeed, using the Gene Ontology annotations of differentially expressed genes, we highlighted differentially expressed genes implicated in energy metabolism or involved in cellular defenses against reactive oxygen species and associated injuries [15]. The use of this tool could therefore offer a new perspective to elucidate the remarkable adaptation of penguins to their environment.

The MAXRS method enabled us to extract biological information even though the global fluorescence intensity signal on our microarrays was low. Candidate genes were highlighted, and the direction of expression variation of 90% of these genes was confirmed by qPCR. This shows that gene expression analysis in species genetically distant from model organisms is possible with heterologous hybridization and an appropriate analysis method. As there are very few transcript sequences available in the penguin, we could not quantify the extent to which our results were affected by cross-hybridization. For this reason, we consider heterologous hybridization as a first step of gene expression analysis, a step that allows us to highlight candidate genes that must be validated by another, complementary method. However, the high rate of validation of our results by qPCR is promising and shows that, even if cross-hybridization should affect our results, this effect should be slight. This method could be useful to analyze microarray results for species highly diverged from the reference species and for those without any sequence data, such as models used in ecophysiology. Finally, we think that this approach is still relevant despite the recent development of next-generation sequencing technologies and RNA-Seq. Indeed, without any reference genome, the RNA-Seq data must be *de novo *assembled, and this is a difficult challenge, as the level of coverage varies greatly between transcripts with different expression levels [16].

## Conclusions

We present MAXRS, a new method to analyze heterologous hybridization on microarrays. This method enabled us to analyze the transcriptome of king penguin by using microarrays dedicated to the chicken. Despite the large phylogenetic distance between these two bird species, we identified differentially expressed genes in the pectoralis muscle of king penguin during the transition from terrestrial to marine life, and we confirmed 90% of the tested gene variations by quantitative PCR. These results are promising for the use of microarray technology in species genetically distant from laboratory models. It will be valuable to transfer this technology to biological fields dealing with non-traditional model organisms, like ecological physiology.

## Methods

### Assessment of sequence divergence between penguin and chicken

For this purpose, we used all *Aptenodytes patagonicus *mRNA sequences available in GenBank. For each of these sequences, we identified the most similar gene family in the Hovergen homologous gene families database [17]. We then replaced the penguin sequence in the phylogenetic tree of the family, which allowed us to identify the putatively orthologous chicken sequence, if available. This analysis was performed with HoSeqI [18]. We then aligned the penguin and chicken sequences by using the water EMBOSS tool [19]. The GenBank accession number of penguin sequences, the accession number of their orthologous chicken sequences, the corresponding Hovergen family identification number and the percent identity between each pair of sequences are available in Additional file 1.

### Animals

Penguin muscle samples were collected at the Crozet archipelago (French Southern Territories) during the austral summer (December 2005 to March 2006), following the ethical recommendations granted by the Ethics Committee of the French Polar Research Institute (IPEV) and by the French Ministry of Environment.

Two sets of juvenile king penguins (*Aptenodytes patagonicus*) were captured according to their degree of acclimation to marine life. In the first group, four penguins were captured just before they underwent their first immersion to cold sea water (called NI for never-immersed), while the second group was composed of three penguins that had completely accomplished their acclimation to marine life (called SA for sea-acclimated). Penguins were anesthetized by isoflurane inhalation, and approximately 100 mg of pectoralis muscle was surgically excised, frozen in liquid nitrogen and kept at -80°C until molecular analysis. At the end of the experiment, birds were monitored for a few days and then released at the site of their capture.

### Microarray analysis

Total RNA was extracted using the TriReagent procedure (Invitrogen, Cergy Pontoise, France) following the manufacturer's instructions. The quality of extracted RNA was assessed using a Bioanalyzer 2100 (Agilent technologies, Inc, Palto Alto, CA, USA). RNA integrity numbers of all samples were greater than 8.

Labeling and hybridization were performed following the Affymetrix protocol [20] using the ProfileXpert platform (Lyon, France) on Affymetrix GeneChip^® ^Chicken Genome Arrays.

### Heterologous hybridization analysis

The MAXRS method we developed for the analysis of heterologous hybridization profiles is described in the Results section.

After using the MAXRS method, results among microarrays were normalized using the quantile method [21], and the test for differential expression between the two conditions was performed with the empirical Bayes moderated t-statistics implemented in the Bioconductor package limma [11]. The resulting p-values were then adjusted for multiple testing by using the Benjamini and Hochberg method [12] implemented in the Bioconductor package multtest. All analyses were done using the R statistical software.

The raw and processed microarray data are available in the Gene Expression Omnibus [22]. For each probe set, the mean intensity value of each probe with an intensity above background and its rank are available in Additional file 4.

### Validation of microarray data analysis using real-time PCR

Quantitative PCR was performed using the same RNA samples to test the accuracy of our analysis. For each sample, 1 μg of total RNA was converted into double-stranded cDNA using 200 U of RT-MMLV reverse transcriptase (Invitrogen, Cergy Pontoise, France), 100 ng of random primers, 1 mM deoxyribonucleotides and 40 U RNase inhibitor, according to the manufacturer's instructions. Then, real-time PCR was performed in a MyiQ thermal cycler (Bio-Rad, Marnes La Coquette, France) using IQ SYBR Green Supermix (Bio-Rad). We designed primers specific to the chicken sequence recognized by the microarray for 15 genes (see Additional file 5) using the information available on the NetAffx website [20] and Primer3 software [23]. To confirm our results with penguin-specific primers, we sequenced the PCR products, designed primers specific to the penguin sequences (see Additional file 5) and performed qPCR using these new primers.

We used the following qPCR conditions: 3 min at 95°C, followed by 40 cycles of denaturation for 10 s at 95°C and annealing/extension for 45 s at 60°C, according to the manufacturer's instructions. All samples were run in duplicate along with dilutions of known amounts of target sequence to quantify the initial cDNA copy number (Concentration = Efficiency^ΔCt^). The results are expressed as the ratio of the target gene over 18 S rRNA concentration (ng/μg) [which was verified to exhibit non-significant variation between the two groups of cDNAs using REST 2009 software (0.29 < p < 0.70)] [24].

## Authors' contributions

CDE and BR carried out all the experiments of this study. CK designed and implemented the MAXRS method. FF helped establish the qPCR quantification. CG, MD and PC provided helpful comments regarding this study and the manuscript. PJ provided facilities and technical support for the field experiments. CDE, CK and BR wrote the manuscript, and CG, CDU and MD corrected it. All authors read and approved the final manuscript.

## Supplementary Material

Additional file 1 **Penguin and chicken orthologous sequences**. GenBank accession numbers of penguin sequences together with the accession numbers of their orthologous chicken sequences, the corresponding Hovergen family identification number and the percent identity between each pair of sequences. Click here for file

Additional file 2 **Comparison of the gene expression differences between qPCR using primers designed against chicken and against penguin transcript sequences**. Expression fold changes of the six genes tested by quantitative PCR using primers designed against chicken (black bars) vs. penguin sequences (gray bars). These fold changes correspond to SA/NI for the genes up-regulated during the transition from terrestrial to marine life (represented above the x-axis) and to NI/SA for the down-regulated genes (represented below the x-axis). Click here for file

Additional file 3 **Comparison of the gene expression differences assessed by GCOS analysis and by qPCR**. Expression fold changes of the six differentially expressed genes determined with GCOS and with qPCR. These fold changes correspond to SA/NI for the genes up-regulated during the transition from terrestrial to marine life (represented above the x-axis) and to NI/SA for the down-regulated genes (represented below the x-axis). The white bars correspond to the fold changes assessed by microarray and analyzed with GCOS, and the black bars correspond to the fold changes assessed by quantitative PCR. Click here for file

Additional file 4 **Mean intensity value and rank of each probe with an intensity above background**. This file provides, for each Affymetrix probe above background, the mean intensity value and rank. Click here for file

Additional file 5 **Primer sequences used for qPCR**. This file provides, for each tested gene, the corresponding Affymetrix probe set ID, the primer sequences used for qPCR and the fold changes and p-values from the microarray and qPCR. Click here for file
