# Supplementary figures and images for: Transcriptome analysis in non-model species: a new method for the analysis of heterologous hybridization on microarrays
Source: BMC Genomics. 2010 May 31;11:344. doi: 10.1186/1471-2164-11-344 (PMC2901317; doi:10.1186/1471-2164-11-344)

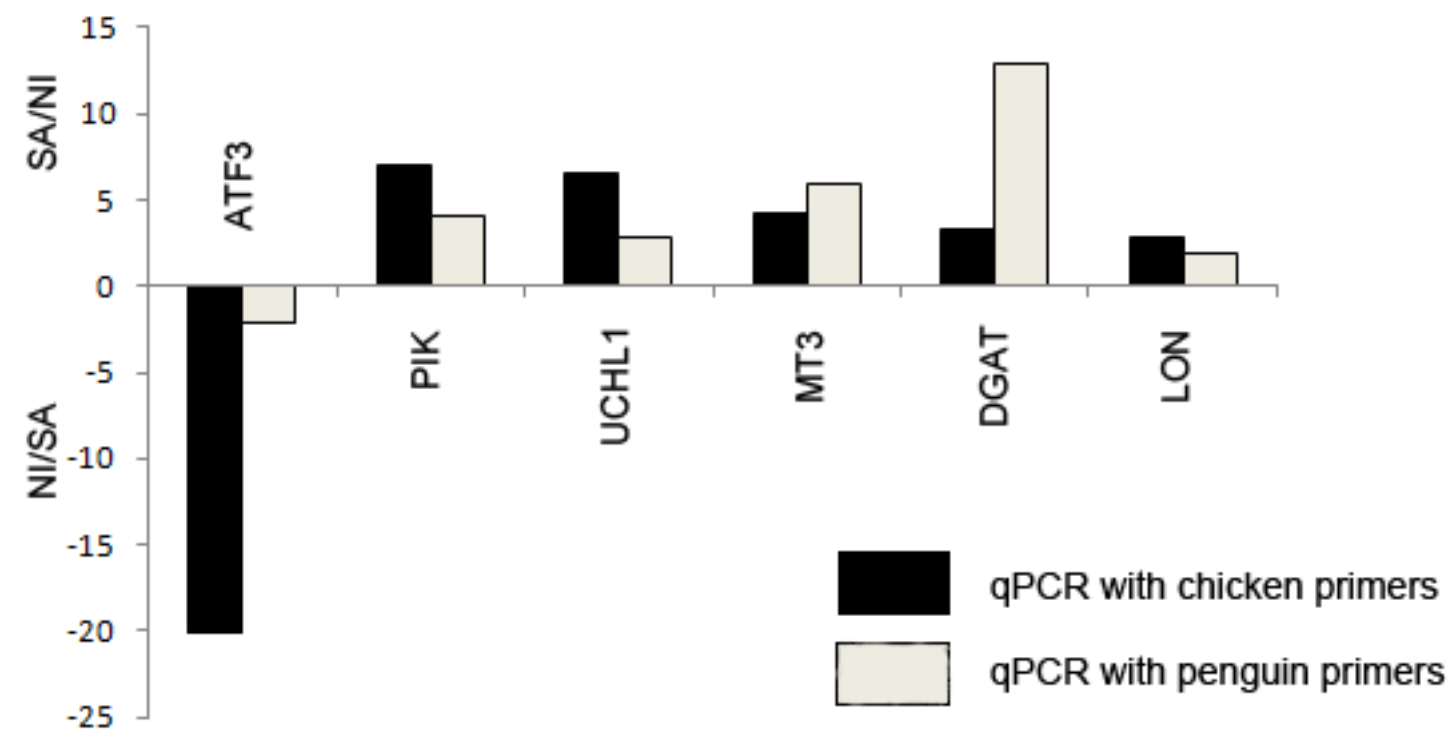

Supplement: Additional file 2 — Comparison of the gene expression differences between qPCR using primers designed against chicken and against penguin transcript sequences. Expression fold changes of the six genes tested by quantitative PCR using primers designed against chicken (black bars) vs. penguin sequences (gray bars). These fold changes correspond to SA/NI for the genes up-regulated during the transition from terrestrial to marine life (represented above the x-axis) and to NI/SA for the down-regulated genes (represented below the x-axis). [file 1471-2164-11-344-S2.PDF]

SA/NI

NI/SA

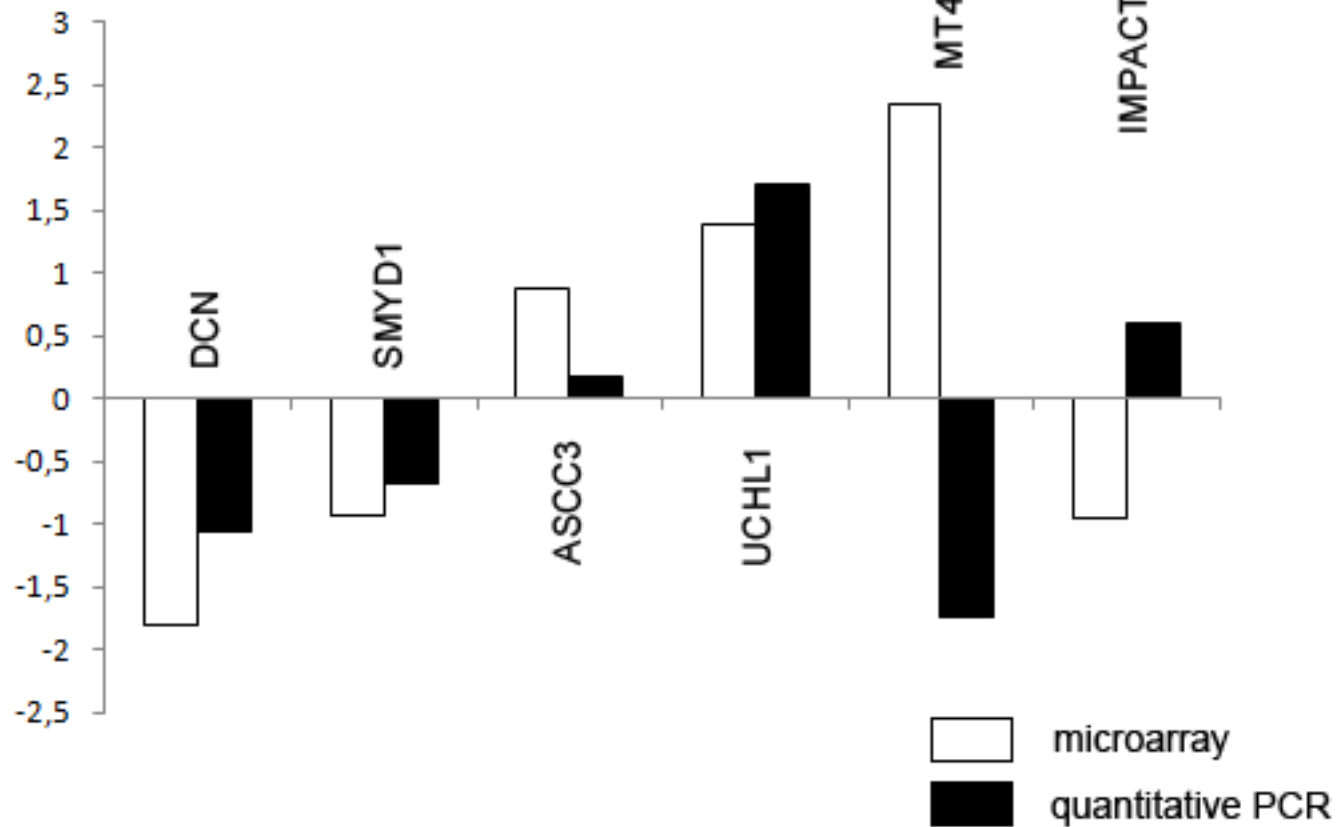

Supplement: Additional file 3 — Comparison of the gene expression differences assessed by GCOS analysis and by qPCR. Expression fold changes of the six differentially expressed genes determined with GCOS and with qPCR. These fold changes correspond to SA/NI for the genes up-regulated during the transition from terrestrial to marine life (represented above the x-axis) and to NI/SA for the down-regulated genes (represented below the x-axis). The white bars correspond to the fold changes assessed by microarray and analyzed with GCOS, and the black bars correspond to the fold changes assessed by quantitative PCR. [file 1471-2164-11-344-S3.PDF]
